# Enhanced Functional Recovery in MRL/MpJ Mice after Spinal Cord Dorsal Hemisection

**DOI:** 10.1371/journal.pone.0030904

**Published:** 2012-02-13

**Authors:** Sandrine Thuret, Michaela Thallmair, Laura L. Horky, Fred H. Gage

**Affiliations:** Laboratory of Genetics, The Salk Institute for Biological Studies, La Jolla, California, United States of America; Columbia University, United States of America

## Abstract

Adult MRL/MpJ mice have been shown to possess unique regeneration capabilities. They are able to heal an ear-punched hole or an injured heart with normal tissue architecture and without scar formation. Here we present functional and histological evidence for enhanced recovery following spinal cord injury (SCI) in MRL/MpJ mice. A control group (C57BL/6 mice) and MRL/MpJ mice underwent a dorsal hemisection at T9 (thoracic vertebra 9). Our data show that MRL/MpJ mice recovered motor function significantly faster and more completely. We observed enhanced regeneration of the corticospinal tract (CST). Furthermore, we observed a reduced astrocytic response and fewer micro-cavities at the injury site, which appear to create a more growth-permissive environment for the injured axons. Our data suggest that the reduced astrocytic response is in part due to a lower lesion-induced increase of cell proliferation post-SCI, and a reduced astrocytic differentiation of the proliferating cells. Interestingly, we also found an increased number of proliferating microglia, which could be involved in the MRL/MpJ spinal cord repair mechanisms. Finally, to evaluate the molecular basis of faster spinal cord repair, we examined the difference in gene expression changes in MRL/MpJ and C57BL/6 mice after SCI. Our microarray data support our histological findings and reveal a transcriptional profile associated with a more efficient spinal cord repair in MRL/MpJ mice.

## Introduction

Injury to the mammalian spinal cord typically results in very limited regeneration of damaged axons, causing permanent functional deficits [1]. Central nervous system (CNS) axonal regeneration appears to be impeded partly by myelin inhibitors [2] and by formation of a post-lesion scar barrier [3]. However, if axons can traverse the injury site, there is evidence that they may re-grow into unscarred regions [4], [5].

The main components of the scar are gliotic astrocytes and microglia/macrophages [6]. Upon injury, astrocytes proliferate, up-regulate the expression of glial fibrillary acidic protein (GFAP) and form a dense network of glial processes at the injury site. In addition to creating this physical barrier, astrocytic gliosis will result in the production of a variety of cytokines, cell adhesion and extra-cellular matrix molecules. Some of these molecules, such as chondroitin sulfate proteoglycans (CSPG), have been shown to inhibit axonal growth [7] and, if degraded in the glial scar at the injury site, axonal regeneration as well as functional recovery is promoted [8]. Similarly, suppression of glial scar formation with a 2-Gy dose of radiation after SCI improves functional recovery [9].

Wound healing of mammalian tissue is a long process and is always accompanied by scarring. In general, adult mammals are poor healers compared to lower vertebrates such as some tailed amphibians, which can completely regenerate their spinal cord without scar formation [10]. Among mammals, the MRL/MpJ mouse strain has a unique capacity for wound healing and regeneration, as shown by the closure of ear punches with normal tissue architecture and cartilage replacement. This characteristic is in contrast to the C57BL/6 mouse, which heals only part of the ear punch hole and forms a scar [11]. Furthermore, the MRL/MpJ mouse has been shown to regenerate injured heart tissue in contrast to the C57BL/6 mouse [12]. Neither of these regenerative repair processes is normally seen in adult mice, and the healing response of MRL/MpJ mice is reminiscent of amphibian regeneration with scarless healing [11], [12]. More recently, Chadwick and colleagues [13] showed that, after amputation, MRL mouse digit tips were found to distally re-grow more quickly and reform nails either partially or completely to a greater degree than C57BL/6 mice. Gourevitch and colleagues [14] further observed a blastema-like structure along apparent chondrogenesis, consistent with a histological profile of a regenerative response to injury.

Because the MRL/MpJ mouse has shown enhanced healing abilities without scarring, we investigated whether injured MRL/MpJ spinal cord would show enhanced healing and possibly axon regeneration without glial scar formation. To assess putative regenerative repair in the MRL/MpJ spinal cord, we performed a dorsal spinal cord hemisection and used C57BL/6 mice as control animals.

## Materials and Methods

### Animals

The MRL/MpJ is an inbred mouse derived from the interbreeding of 4 progenitor inbred lines: LG/J (75%), AKR/J (12.6%), C3H/Hej (12.1%) and C57BL/B6 (0.3%) [15]. Over 20 genetic loci have been identified and their healing phenotype is a complex multigenic and sexually dimorphic trait [16], [17], [18], [19].

We chose C57BL/6 animals as controls because they have been used as a control strain in all published work related to the enhanced healing abilities of MRL/MpJ mice, including all gene and loci identification studies [16], [17], [18], [19], [20], [21]. Using the C57BL/6 mice as a control strain thereby allows us to compare our gene expression data to already published literature. The intact C57BL/6 mice performed better than the MRL/MpJ animals in the rotarod and the grid test. Therefore, the motor recovery results were analyzed after normalizing the performance of the injured MRL/MpJ and C57BL/6 mice to their respective baseline levels (intact mice). It is likely that the MRL/MpJ mice are not as agile as the C57BL/6 mice because of their higher body weight (10-week-old female C57BL/6 weighed 20.1 g±0.5 g, MRL/MpJ weighed an average of 31.7 g±0.4 g). The only available inbred strains of mice matching the weight of MRL/MpJ mice, the C3HeB/FeJ strain, is blind (Mouse Phenome Database, http://www.jax.org) and therefore would not have been suitable for behavioral testing.

Ten-week-old female MRL/MpJ and C57BL/6 mice were purchased from the Jackson Laboratory, ME. They were housed in groups of 5 or singly (after SCI) and had access to water and food ad libitum at the Salk Institute animal facilities. All animal procedures were in accordance with NIH guidelines and approved by the Salk Institutional Animal Care and Use Committee. Intraperitoneal injections of ketamine (100 mg/kg) and xylazine (10 mg/kg) were used as anesthesia for all surgical procedures and before perfusion.

### SCI and post-operative care

Under appropriate aseptic techniques, 10-week-old female mice (22 MRL/MpJ and 22 C57BL/6) received a partial dorsal laminectomy at a single thoracic vertebral segment (T9). The spinal cord was exposed and Lidocaine (2%, 10 µl, Bimeda, Riverside, MO) was applied to the dura for 1 minute to anesthetize the region to be transected. The dorsal half of the spinal cord was cut 0.5 mm deep with a pair of microscissors to sever the main parts of the CST, including the dorsal lateral component of the CST (the diameter of the spinal cord at T9 was not significantly different between the two strains, allowing injuries of equal sizes for both strains). The overlaying muscle layers were sutured and the skin was stapled closed. Postoperatively, all animals were placed singly in a cage on warming pads and were provided with moist food pellets on the bottom of the cage. Temperature and respiration were monitored until animals were fully awake. Then cages were placed back on the housing system rack. Bladders were manually expressed twice a day until normal voiding reflexes returned. An antibiotic (enrofloxacin, 2.27 mg/kg, Baytril, Bayer, KS) was given once daily for 6 days. Animals were transcardially perfused with saline, followed by 4% paraformaldehyde at different time points post-SCI.

### Behavioral analyses

Intact MRL/MpJ and C57BL/6 mice (n = 10 in each group) were used to establish the baseline performance of each strain in the rotarod, grid and footprint test (baseline group). Also, injured groups of 10 MRL/MpJ and 10 C57BL/6 mice were tested 4 days before the injury and up to 88 days after the injury.

#### Rotarod test

The Rotamex 4/9 (Columbus Instrument, OH) was used to evaluate fore-and hindlimb motor coordination and balance. Each mouse was placed on the slowly moving rotarod (5 rpm) and the trial was started when the animal found its balance. The rotarod accelerated from 5 rpm to a maximum of 70 rpm within 3 minutes. The latency to fall off the rotarod was recorded. Mice underwent 3 consecutive trails. The mean latency for 3 trails was used for statistical analysis.

#### Grid test

This test evaluated the ability of the mice to navigate over a wire mesh grid (2.5×2.5 cm grid spaces, 28×35 cm total area). Each mouse was videotaped while on the grid until it achieved a total of 20 steps. A foot-fault was scored each time the animal misplaced its hindlimb to protrude entirely or partially through the grid.

#### Bladder function

The bladder of each injured animal was voided twice daily until it recovered autonomic bladder function. The amount of urine voided (or the level of “bladder fullness”) was estimated and ranked from 3 to 0: 3 = dysfunctional; full bladder, medium to high pressure needed to manually express it and a large amount of urine expressed, 2 = medium amount of urine expressed after medium pressure, 1 = small amount of urine expressed after slight pressure, and 0 = empty bladder and no urine expressed.

### CST tracing

Six MRL/MpJ and 6 C57BL/6 received bilateral CST tracing at 40 days post-injury (dpi), 10 MRL/MpJ and 10 C57BL/6 at 95 dpi, and 2 intact MRL/MpJ and 2 intact C57BL/6 also received bilateral CST tracing. A hole was drilled on each side of the skull overlying the sensorimotor cortex. The anterograde neuronal tracer biotin dextran amine (BDA) (10% BDA in 0.01 M phosphate buffer, pH 7.4; Molecular Probes, Eugene, OR) was injected (2 µl) at 4 injection sites into the sensorimotor area (anterior-posterior coordinates from bregma in mm: 1.0/1.5, 0.5/1.5, −0.5/1.5, −1.0/1.5, at a depth of 0.5 mm) using a Hamilton syringe. Two weeks after BDA injection, the animals were perfused and tissue was collected for histology.

### BrdU injections

Bromodeoxyuridine (BrdU; Sigma) was dissolved in 0.9% NaCl and filtered at 0.2 µm. To label dividing cells in intact mice, 12 animals received a daily intraperitoneal injection for 6 consecutive days at a dose of 50 mg/kg body weight (10 mg/ml BrdU). Six mice were sacrificed 1 day and 6 mice were sacrificed 4 weeks after the last injection to assess rate of proliferation, differentiation and survival of mitotic cells. SCI mice received the first injection 2 hours after the injury. Six lesioned mice received a single injection 2 hours after injury and were sacrificed 1 day later. Twenty injured mice received one injection per day for 6 days. Of these, 10 animals were sacrificed at 54 dpi and 10 were sacrificed at 109 dpi.

### Tissue processing

Spinal cords were cut into 7-mm segments and embedded in cryomolds (Fisher Scientific, Pittsburgh, PA) with O.C.T. mounting medium (Tissue Tek, Torrance, CA). Mounted spinal cord segments were stored at −80°C until processed further. Spinal cords were sectioned sagittally on a cryostat at 20 µm thickness and stored on slides at −20°C.

### CST detection and analysis

The spinal cord sections were incubated with an avidin–biotin–peroxidase complex (ABC Elite, Vector Laboratories, Burlingame, CA), and the BDA tracer was visualized by a nickel-enhanced diaminobenzidine (DAB) reaction [22]. BDA-labeled fibers were quantified at 0.5 mm and 2 mm caudal and at 5 mm rostral to the lesion site. To account for inter-individual tracing variability, numbers were normalized to the amount of fibers 5 mm rostral to the lesion site, where the CST was intact, and are presented as percentages [8].

### BrdU immunohistochemistry and quantification

For the stereological quantification of BrdU-labeled cells, cryostat sections were stained with rat anti-BrdU primary antibody (1∶100; Accurate Chemicals) for DAB immunohistochemistry. BrdU-labeled cells in intact mice were quantified as described before [23]. To obtain the number of BrdU-labeled cells per mm^3^ in injured spinal cords, labeled cells were counted at the injury epicenter, every 60 µm through the entire width of the spinal cord. For each sagittal section, the area surrounding the injury epicenter (5 mm surrounding the injury epicenter) was traced and recorded using a mechanical stage attached to an Olympus BH2 microscope, a Dage MTI CCD-300TIFG Video camera (Michigane City, IN) and the StereoInvestigator software (MicroBrightfield, Colchester, VT). The number of labeled cells within each traced area was recorded.

### Multiple marker immunofluorescence and quantification

Sections were processed for multiple markers to determine the phenotype of BrdU-labeled cells. Primary antibodies detecting immature and mature astrocytes (S-100β polypeptide), mature astrocytes (GFAP), oligodendrocyte progenitors (NG2), microglia (OX-42), and immature (TUJ1) and mature neurons [neuronal nuclear antigen clone A60 (NeuN)] were used. Cryostat sections were pretreated for BrdU detection as described previously [23]. Three compatible primary antibodies were incubated on the sections for 2 days at 4°C in TBS +0.1% Triton X-100 +5% donkey serum. Primary antibodies were used at the following concentrations: rat anti-BrdU (1∶100; Accurate Chemicals), rabbit anti-S-100β (1∶10,000; SWant, Bellinzona, Switzerland), mouse anti-NeuN (1∶10; clone A60; Dr. R. Mullin, Salt Lake City, UT), rabbit anti-GFAP (1∶1000; Dako, Carpinteria, CA), rabbit anti-NG2 (1∶500; Chemicon, Temecula, CA) and mouse anti-OX-42 (1∶200; Serotec, UK). Sections were rinsed twice in 0.1 M TBS, pH 7.5, and blocked in 0.1 M TBS +0.1% Triton X-100 for 15 min before application of secondary antibodies. The following secondary antibodies were applied in 0.1 M TBS and 0.1% Triton X-100, each at a concentration of 1∶250 for 2 hr in the dark: donkey anti-rat IgG conjugated to FITC (1∶250; Jackson ImmunoResearch, West Grove, PA), donkey anti-mouse conjugated to Cy2 or Cy5 (1∶250; Jackson ImmunoResearch), and donkey anti-rabbit conjugated to Cy2 or Cy5 (1∶250; Jackson ImmunoResearch). Incubation with secondary antibodies was followed by three rinses (15 min) in TBS. Slides were then immediately coverslipped using polyvinyl alcohol-1,4 diazabicyclo[2.2.2]octane and kept in the dark at 4°C until analysis.

Multiple label immunofluorescent images were collected and quantified using confocal microscopy (Bio-Rad MRC 1000, Hercules, CA). Single confocal plane images of BrdU or phenotype markers (see above) were collected and combined to assess co-localization. A cell was scored for double-staining when a well-defined BrdU-labeled nucleus was associated with an immunopositive (e.g., GFAP, S100b etc.) cell body. The entire cell was followed through the *z*-axis, and only cells with a well-circumscribed, immunopositive soma were considered positive for a particular phenotype. A total of 100 BrdU cells were randomly counted at the injury epicenter.

### TdT-mediated dUTP nick end labelling (TUNEL) assay

Apoptosis was examined by TUNEL assay, using a commercially available kit (Oncor, Apoptag). The modified protocol developed by Whiteside et al. was followed to simultaneously counterstain with Hoechst 33342 [24]. TUNEL positive cells were only counted as apoptotic cells if the nucleus had fragmented into several membrane-bounded, highly condensed bodies and if it exhibited shrinkage of both cytoplasm and nucleus, creating pericellular space. Any artifacts of TUNEL labeling, i.e., non-nuclear material, was disregarded.

### Detection and quantification of micro-cavities

The sections were counterstained with GFAP antibody (rabbit anti-GFAP, 1∶1000; Dako). To obtain the number of micro-cavities per mm^3^, micro-cavities were counted at the injury epicenter, every 60 µm through the entire width of the spinal cord. For each section the area surrounding the injury epicenter was traced and recorded as described above. The number of micro-cavities within the traced area was recorded.

### Statistical evaluation

All the behavioral data and quantification of traced CST fibers were analyzed using a two-factor ANOVA. Post hoc analysis was carried out using Bonferroni-corrected individual comparisons (Microsoft Excel and XLSTAT; Addinsoft, NY). Statistical evaluation of bladder recovery was assessed by a non-parametric Mann Whitney test. Statistical evaluation of BrdU cell counts and micro-cavity quantification was performed using a Student's t-test. In all analyses, a P-value of less than 0.05 was chosen as significance threshold. All data are presented as the mean ± standard error of the mean (SEM).

### Experimental design for the microarray gene expression analysis

For MRL/MpJ and C57BL/6 mice, three groups were examined at 4 dpi: intact control, laminectomy control and hemisection at T9. For each condition, 3 mice were sacrificed and a 1-cm spinal cord segment containing the injury epicenter was collected.

### RNA extraction and microarray procedures

The procedure was performed as described in detail before [25]. Total RNA was extracted from each sample individually using TRIzol reagent (Invitrogen, Carlsbad, CA). First-strand cDNA was generated by using a T7-linked-(dT)_24_ primer. After second-strand synthesis, the double-stranded cDNA was used for in vitro transcription using the ENZO BioArray High-Yield RNA transcript labeling kit (ENZO, Farmingdale, NY). The labeled cRNA was purified and fragmented. Each fragmented, biotin-labeled cRNA sample (20 µg) was hybridized to an Affymetrix mouse 430A chip. After scanning, array images were assessed by eye to confirm scanner alignment and the absence of significant bubbles or scratches. Chips had to meet the following criteria to be included in the data analysis: the number of probe pairs called present had to be greater than or equal to 30%, all spiked-in bacterial controls had to be called present, background had to be <200, the number of outliers had to be <500, and the 3V/5V ratio of GAPDH and actin had to be <2.

### Microarray data analysis and quantitative PCR validation

The chips were analyzed using two different complementary analytical tools. Data were preprocessed (*.dat files to *.cel files) using Affymetrix Microarray Analysis Suite (MAS) 4.0. Data were subsequently analyzed using the dChip 1.3 PM-MM [26] and the Drop Method [27]. dChip approximates expression values by modeling the whole body of chip data to an ideal system, and Drop determines the significance of change between the triplicate groups statistically with minimal assumptions.

To detect up- or down-regulated genes following SCI, Hemisection chips were compared to Laminectomy control chips for both strains of mice. Each group had 3 chips, so each comparison had a 3×3 design, with a possibility of 9 pairwise comparisons for each group. To obtain a list of genes that were differentially expressed after SCI in MRL/MpJ animals, but not significantly changed in C57BL/6 mice, we compared the gene lists of MRL/MpJ and C57BL/6 mice after imposing very strict criteria (dChip fold change>1.2 AND Drop method confidence = 95%) for the MRL/MpJ chips and very loose criteria (dChip fold change>1.2 OR Drop method confidence = 50%) for the C57BL/6 chips. To detect differentially expressed genes between the 2 uninjured strains, Intact control chips from MRL/MpJ were compared to Intact control chips from C57BL/6. For statistical significance we imposed dChip fold change>1.2 and Drop method confidence = 95%.

Expression changes of genes of interest were verified by quantitative RT-PCR (Q-PCR). cDNA was prepared from RNA samples used for GeneChip analysis, and reverse transcription was performed using Superscript First-strand cDNA Synthesis System (Invitrogen). Primer Express v1.5 software was used to design oligonucleotide primers (Applied Biosystems, Foster City, CA) (see methods Table S1 for sequences of primers), which were constructed by Sigma-Aldrich. Q-PCR was performed with 3.33 ng of cDNA, SYBR Green Master Mix (Applied Biosystems), and 0.2 µM of each primer in a 25-µl reaction. All reactions were performed in triplicate using an ABI Prism 7700 Sequence Detection System (Applied Biosystems). Data analysis was performed according to the protocol provided by Applied Biosystems using Sequence Detector Systems v1.7 software. Intact spinal cord cDNA was used to create standard curves for both strains. Expression of each gene was calculated based on the standard curve for a given primer set. The relative amount of calculated message was normalized to the level of a control gene (18s rRNA). The Q-PCR results are summarized in table 1

**Table 1:** Fold changes for the 10 most up- and down-regulated genes in MRL/MpJ compare to C57BL/6 mice after spinal cord hemisection.

Up-regulated Genes	MRL/MpJ Fold Change	C57Bl/6 Fold Change	Down-regulated Genes	MRL/MpJ Fold Change	C57Bl/6 Fold Change
interferon activated gene 202B [GeneID: 26388]	35.66 [48.80]	1.00 [0.98]	Mus musculus adult male olfactory brain cDNA, RIKEN full-length enriched library, clone:6430537M22 product:unknown EST, full insert sequence.	−4.43	−1.82
allograft inflammatory factor 1 [GeneID: 11629]	23.83 [32.15]	3.14 [2.32]	potassium voltage-gated channel, shaker-related subfamily, beta member 1 [GeneID: 16497]	−3.75 [−4.52]	−1.61 [−2.96]
histidine ammonia lyase [GeneID: 15109]	18.4 [15.34]	2.09 [3.43]	calcitonin-related polypeptide, beta [GeneID: 116903]	−3.55 [−4.82]	−1.50 [−1.03]
Mus musculus, clone MGC:28142 IMAGE:3982042, mRNA, complete cds	7.88	2.70	vesicle-associated membrane protein 1 [GeneID: 22317]	−3.41 [−2.98]	−1.34 [−0.63]
cDNA sequence BC032204 [GeneID: 108101]	5.75	1.85	sulfotransferase family 4A, member 1 [GeneID: 29859]	−3.36 [−4.23]	−1.63 [−1.06]
apolipoprotein B editing complex 1[GeneID: 11810]	4.68 [6.98]	1.68 [1.85]	regulator of G-protein signaling 4 [GeneID: 19736]	−3.16 [−2.98]	−1.35 [−0.85]
cDNA sequence BC032204 [GeneID: 108101]	4.45	1.81	Parvalbumin [GeneID: 19293]	−3.10 [−4.50]	−1.32 [−1.28]
Rho, GDP dissociation inhibitor (GDI) beta [GeneID: 11857]	4.44 [3.96]	1.85 [1.78]	creatine kinase, mitochondrial 1, ubiquitous [GeneID: 12716]	−3.02 [−2.20]	−1.87 [−1.00]
beta-glucuronidase [GeneID: 110006]	4.27 [3.98]	1.63 [1.86]	cartilage acidic protein 1 [GeneID: 72832]	−3.00 [−5.02]	−1.30 [−1.32]
cysteine rich protein 61 [GeneID: 16007]	4.18 [6.40]	1.86 [2.59]	solute carrier family 17 (sodium-dependent inorganic phosphate cotransporter), member 6 [GeneID: 140919]	−2.94 [−1.98]	−1.23 [−0.75]

The complete data set is available at http://genechip.salk.edu/rawdata/Thuret_MRL.zip.

Expression changes of genes of interest were also verified by quantitative RT-PCR (Q-PCR). When applicable, the Q-PCR fold changes are presented in brackets [ ].

## Results

### Enhanced functional recovery of MRL/MpJ mice after SCI

The locomotor performance of injured mice was assessed at several time points up to 88 dpi. Due to the disparity in performance between intact MRL/MpJ and C57BL/6 mice, a control group of intact, age-matched MRL/MpJ and C57BL/6 animals underwent the same behavioral tests as the injured mice to establish the baseline performance for both mice strains.

**Figure 1:**
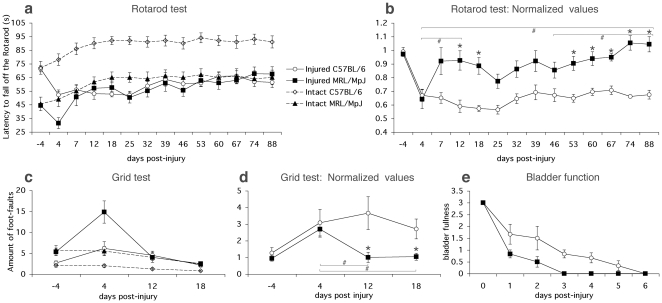
Enhanced functional recovery after SCI in MRL/MpJ mice. **a**, The latency to fall off the rotarod is depicted as a function of time for intact (baseline) and injured MRL/MpJ and C57BL/6 mice. In both strains intact mice improved their performance over the course of the 88 days of training, but the C57BL/6 performed better at all time points tested. The injured MRL/MpJ reached their baseline level by the end of the 88 days of testing, whereas the injured C57BL/6 did not. **b**, Rotarod performance of injured mice is expressed as a ratio of baseline performance. Injured MRL/MpJ performed significantly better than injured C57BL/6 at 12, 18, 53, 60, 67, 74 and 88 dpi (*P<0.002). Injured MRL/MpJ significantly improved between 4 dpi and 88 dpi (#P<0.002), whereas injured C57BL/6 did not improve during that period. **c**, The number of foot-faults made during grid walking is reported as a function of time for intact (baseline) and injured MRL/MpJ and C57BL/6 mice. Both intact strains improved their ability to cross the grid with fewer foot-faults, but intact MRL/MpJ made more foot-faults throughout the entire training period. The injured MRL/MpJ reached their baseline level already by 12 dpi, whereas the injured C57BL/6 still had not reached their baseline level by the end of the testing period at 18 dpi. **d**, Grid walking performance of injured mice is expressed as a ratio to baseline performance. Injured MRL/MpJ performed significantly better than injured C57BL/6 at 12 and 18 dpi (p<0.05). Injured MRL/MpJ had significantly improved between 4 dpi and 12 dpi, 18 dpi (p<0.05), whereas injured C57BL/6 did not improve during that period. **e**, Estimation of bladder fullness is reported as a function of time after SCI in MRL/MpJ and C57BL/6 mice. All MRL/MpJ mice recovered autonomic bladder function by 3 dpi, whereas in C57BL/6 mice, autonomic bladder function did not returned until 6 dpi (Mann Whitney test, p-value = 0.028). Data present mean ± SEM. * denotes significant difference between MRL/MpJ and C57BL/6 and # denotes significant differences in MRL/MpJ performance between time points (P value: two-way ANOVA, Bonferroni post-hoc analysis).

The rotarod test evaluates fore- and hindlimb motor coordination and balance. Intact mice of both strains improved and reached a plateau at 18 days, showing the same performance up to 88 days (Fig. 1a). However, C57BL/6 mice performed better from the start (72.02 s±4.73) and were able to stay longer on the rotarod (at 88 days, 91.1 s±4.5) than the MRL/MpJ (at start: 45.56 s±5.03; at 88 days, 65.1 s±5.4; Fig.1a). In both mouse strains, the rotarod perfomance decreased after SCI (MRL/MpJ, −13.0 s±4.0 and C57BL/6, −19.0 s±3.5). Interestingly, the injured MRL/MpJ mice reached their baseline level by the end of 88 days of testing, whereas the performance of the injured C57BL/6 animals was still reduced (23.5 s±3.9 below their baseline performance at 88 days; Fig. 1a). To eliminate the strain differences in rotarod performance from the recovery curve after SCI, the performance of the injured MRL/MpJ and C57BL/6 mice was normalized to their respective baseline levels (Fig. 1b). There was no difference in the normalized rotarod performance of MRL/MpJ and C57BL/6 mice at 4 days after injury, indicating that both strains were affected equally by the SCI. Interestingly, MRL/MpJ mice recovered rotarod performance over time whereas C57BL/6 did not recover over the 88 days of testing [repeated measures ANOVA: effect of group (F_1, 18_ = 149.2, P<0.0001), time (F_13, 234_ = 5.5, P<0.0001), interaction group×time (F_13, 234_ = 3.1, P<0.0005)]. Individual comparisons (Bonferroni post-hoc) between groups indicated that injured MRL/MpJ performed significantly better than injured C57BL/6 at 12, 18, 53, 60, 67, 74 and 88 dpi (P<0.002).

The grid test was used to assess deficits in descending fine motor control after SCI. Intact animals of both mouse strains improved their ability to cross the grid with fewer foot-faults over time (Fig. 1c). However, intact MRL/MpJ animals made significantly more foot-faults through the entire training period (18 days, 2.4±0.5 foot-faults; p = 0.0025) than C57BL/6 (18 days, 0.8±0.15 foot-faults). Following SCI, animals from both strains made more foot-faults when crossing the grid: MRL/MpJ 9.5±2.7 and C57BL/6 mice 3.5±1.6 additional foot-faults. The injured MRL/MpJ animals reached their baseline level by 12 dpi, whereas the injured C57BL/6 never recovered back to their baseline level within the testing period of 18 days (Fig. 1c). To account for differences in baseline performance, the number of foot-faults of the injured mice was expressed as a ratio of the baseline (Fig. 1d). Again, we did not find significant differences in the normalized performance on the grid walk of MRL/MpJ and C57BL/6 4 days after the injury, indicating that both strains were impaired similarly by the SCI. MRL/MpJ mice showed a better performance than C57BL/6 on the grid at 12 and 18 days [repeated measures ANOVA: effect of group (F_1, 18_ = 10.4, P<0.002), time (F_3, 54_ = 3.7, P<0.01), interaction group×time (F_3, 54_ = 2.0, P>0.1); in individual comparisons (Bonferroni post-hoc), injured MRL/MpJ performed significantly better than injured C57BL/6 at 12 and 18 dpi, P<0.05].

The gait of the lesioned mice was evaluated by analyzing footprint patterns. When assessing base of support and stride length, we found a trend for the injured MRL/MpJ mice to recover faster than the injured C57BL/6 animals, but the differences were not statistically significant (data not shown).

The bladders of the injured animals were manually expressed twice daily until the mice had recovered autonomic bladder function. At 6 hours post-injury (0 dpi), none of the injured animals showed autonomic bladder function. All MRL/MpJ mice recovered autonomic bladder function by 3 dpi, whereas in C57BL/6 mice, autonomic bladder function did not return until 6 dpi [Mann Whitney test of group difference in Area Under Curve summary statistic of mouse bladder function recovery; p-value = 0.028)].

### Enhanced regeneration of CST axons in MRL/MpJ mice after SCI

**Figure 2:**
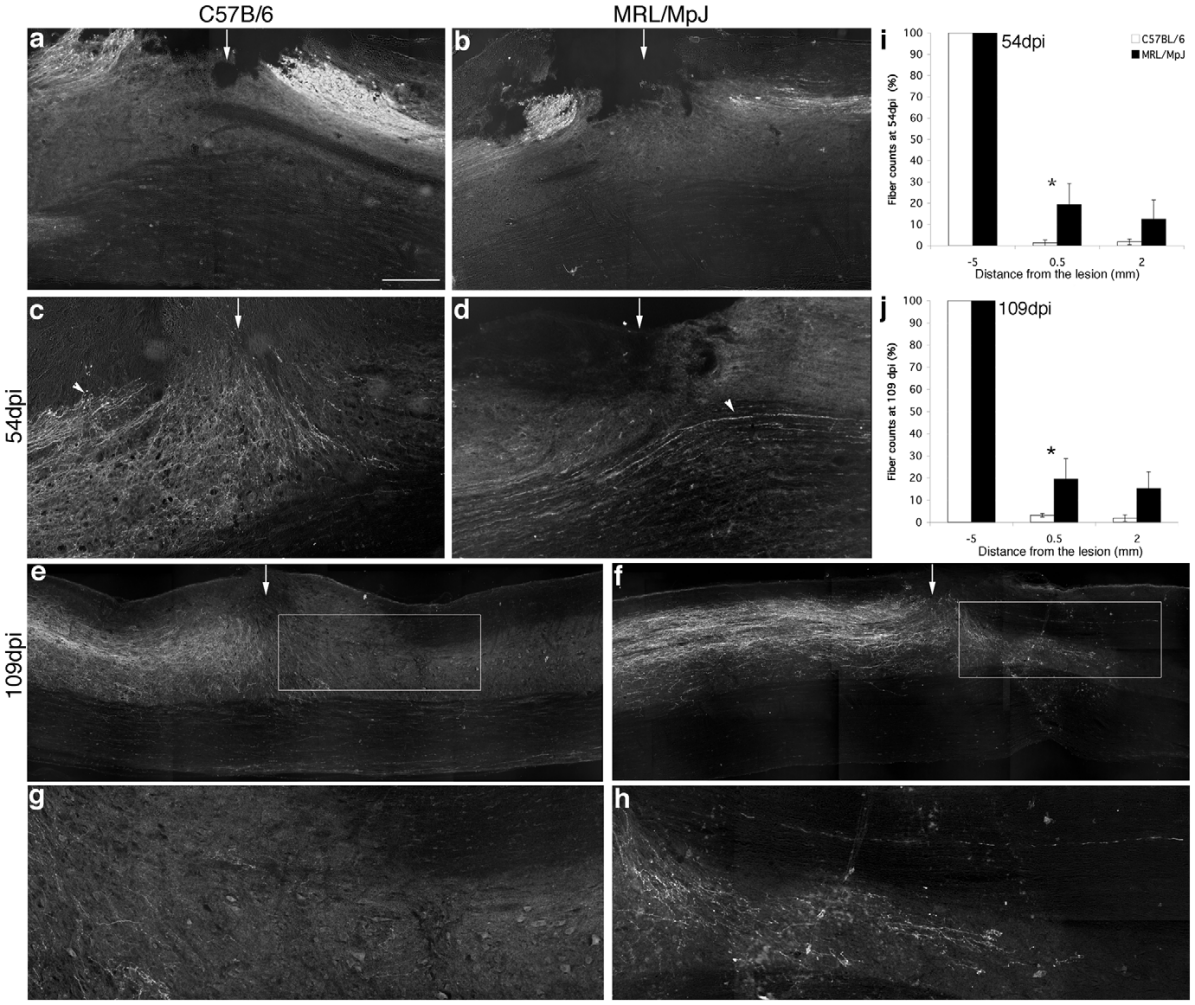
Enhanced regeneration of corticospinal tract (CST) axons in MRL/MpJ mice after SCI. **a**, **b**, Sagittal sections of BDA-labeled CST axons in C57BL/6 (**a**) and MRL/MpJ (**b**) mice. Two weeks after tracing, dorsal hemisections were performed. The CST was analyzed at 4 dpi ( = 18 days after tracing). In both mouse strains, axons are visible rostral and caudal from the injury site. No BDA-labeled fibers were found at the injury site, confirming that the CST was completely transected. **c**, **d**, Sagittal sections of BDA-labeled CST axons in injured C57BL/6 (**c**) and MRL/MpJ (**d**) mice at 54 dpi. Here, BDA tracing was performed right after SCI. C57BL/6 CST axons retracted from the lesion (arrowhead in **c**). In contrast, some MRL/MpJ CST fibers crossed through the lesion site (arrowhead in (**d**)). **e**, **f**, Montage of sagittal sections of BDA-labeled CST axons in C57BL/6 (**e**) and MRL/MpJ (**f**) mice at 109 dpi. Higher magnifications are shown in **g** (from boxed area in **e**) and **h** (from boxed area in **f**). We found no BDA-positive fibers below the injury site in C57BL/6 spinal cord (**e**, **g**), in contrast to MRL/MpJ mice (**f**, **h**). **i**, **j**, Data represent percentage of fibers and are reported as a function of the distance from the lesion at 54 dpi (i) and 109 dpi (j). MRL/MpJ mice exhibited significantly more regenerating axons at 0.5 mm caudal from the lesion site. Rostral end of the spinal cord is to the left. The arrows point at the injury site. Asterisks denote significant differences between MRL/MpJ and C57BL/6, P<0.02 (two-way ANOVA, Bonferroni Post-hoc). Scale bar, 250 µm (**a**–**d**); 300 µm (**e**, **f**); 100 µm (**g**, **h**).

To show that the CST was completely transected by our lesion approach (a 0.5 mm dorsal hemisection), intact MRL/MpJ and intact C57BL6 mice were injected with BDA. Two weeks later these mice underwent a dorsal hemisection and the CST was assessed at 4 dpi (i.e., 18 days after tracing). In both mouse strains the dorsal CST was BDA-labeled and fully transected. Axons were observed rostral as well as caudal to the injury site, but there were no BDA-labeled fibers in the injury epicenter (Fig. 2 a, b).

To analyze the regeneration of the CST after SCI, injured animals were injected with BDA at 40 or 95 dpi. Irrespective of the time point of BDA tracing, we found that C57BL/6 CST axons had retracted from the lesion site and we observed very few or no fibers caudal to the lesion (Fig. 2c, e, g). In contrast, MRL/MpJ CST fibers were present at the lesion site as well as caudal to the injury. Moreover, in some cases, CST axons crossed through the lesion site (Fig. 2d, f, h). At both time points, MRL/MpJ mice displayed significantly more fibers at and caudal to the lesion site compared to C57BL/6 mice [Fig. 2i, j; two-way ANOVA revealed significant effect of group (54 dpi: F_1, 10_ = 6.8, P<0.05; 109 dpi: F_1, 18_ = 11.2, P<0.005)]. Individual comparisons (Bonferroni post-hoc) between groups indicated that injured MRL/MpJ had significantly more regenerating fibers at 0.5 mm rostral to the lesion site (P<0.02): 54 dpi: 19.3±9.9% for MRL/MpJ versus 1.3±1.3% for C57BL/6; 109 dpi: 19.5±9.2% for MRL/MpJ versus 3.2±1.5% for C57BL/6.

In both mouse strains, no significant difference in fiber quantification was found between 54 dpi and 109 dpi, demonstrating that the regenerated fibers were stable and did not retract.

### Reduced astrocytic response and micro-cavity formation in MRL/MpJ mice after SCI

**Figure 3:**
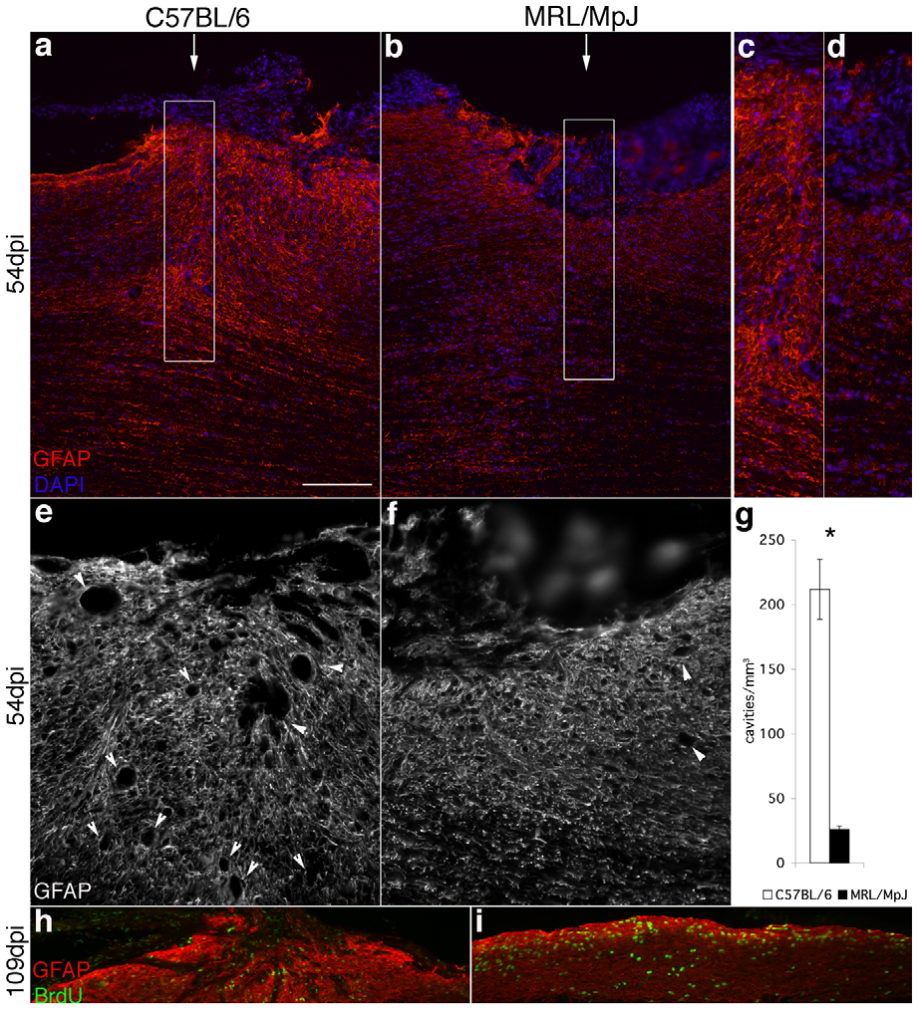
Reduced astrocytic response and micro-cavity formation in MRL/MpJ mice after SCI. **a**, **b**, Sagittal sections of the lesion epicenter at 54 dpi labeled with GFAP (red) and counterstained with DAPI (blue) in C57BL/6 (**a**) and MRL/MpJ (**b**) mice. Higher magnifications are shown in **c** (from boxed area in **a**) and **d** (from boxed area in **b**). The dorsal surface of the injury site in the C57BL/6 mice bulged out and was filled with GFAP-positive astrocytes. In white and gray matter, numerous GFAP-positive astrocytes delineated the incision site (**a**, **c**). In MRL/MpJ mice, the dorsal surface of the injury site was concave and contained GFAP-negative cells surrounded by a few GFAP-positive astrocytes. The incision site was not visible any more (**b**, **d**). **e**, **f**, Sagittal sections of the lesion epicenter at 54 dpi stained for GFAP in C57BL/6 (**e**) and MRL/MpJ (**f**) mice. Numerous micro-cavities (arrowheads) are present at the injury site (arrows) in C57BL/6 mice (**e**) compared to none or few in MRL/MpJ mice (**f**). **g**, Bars represent the number of micro-cavities per cm^3^ at the injury epicenter at 54 dpi. C57BL/6 injured spinal cord developed significantly more micro-cavities than MRL/MpJ cord. **h**, **i**, Sagittal sections of the lesion epicenter at 109 dpi labeled with GFAP (red) and BrdU (green) in C57BL/6 (**h**) and MRL/MpJ (**i**) mice. The injury site of the C57BL/6 mice was filled with GFAP-positive astrocytes and micro-cavities were present (**h**). The injury site in the MRL/MpJ mice was not visible any more and could only be located by the presence of BrdU+ cells. The dorsal surface of the injury site was flat and few GFAP-positive astrocytes were present. (**i**). Asterisks denote significant difference between MRL/MpJ and C57BL/6, P<0.002 (Student's t-test). Scale bar, 250 µm (**a**, **b**, **h**, **i**); 140 µm (**c**, **d**); 125 µm (**e**, **f**).

At 54 dpi the dorsal surface of the injury site in the C57BL/6 mice was convex, bulging and filled with densely packed GFAP-positive astrocytes. Numerous GFAP-positive astrocytes were delineating the injury site forming a glial scar (Fig. 3a, c). In MRL/MpJ mice, the dorsal surface of the injury site was concave and contained GFAP-negative cells surrounded by a few GFAP-positive astrocytes. The incision site itself was no longer visible in MRL/MpJ spinal cord (Fig. 3b, d). Furthermore, the lesion epicenter of C57BL/6 mice contained 10 times more micro-cysts than the lesion site of MRL/MpJ mice (211.9±23.3 micro-cavities/mm^3^ vs. 26.1±2.7 micro-cavities/mm^3^; Student's t-test, p<0.002; Fig. 3.e, f, g).

At 109 dpi the tissue looked similar to 54 dpi in C57BL/6 mice: the injury site in the C57BL/6 mice was still visible, filled with GFAP-positive astrocytes and micro-cavities (Fig. 3h). In MRL/MpJ mice, the dorsal surface of the injury site was flat and fewer GFAP-positive astrocytes were present (Fig. 3i) when compared to the C57BL/6 injury site. The lesion site could be located because of the higher cell division at the injury site compared to the surrounding spinal cord tissue.

### Cell proliferation is different in the intact and injured spinal cord in MRL/MpJ mice

Previous studies have identified progenitor cells throughout the intact and injured rodent spinal cord [23], [28], [29], [30]. Therefore, we examined whether differences between C57BL/6 and MRL/MpJ endogenous spinal cord progenitor cell proliferation could account for the enhanced healing properties of MRL/MpJ mice.

**Figure 4:**
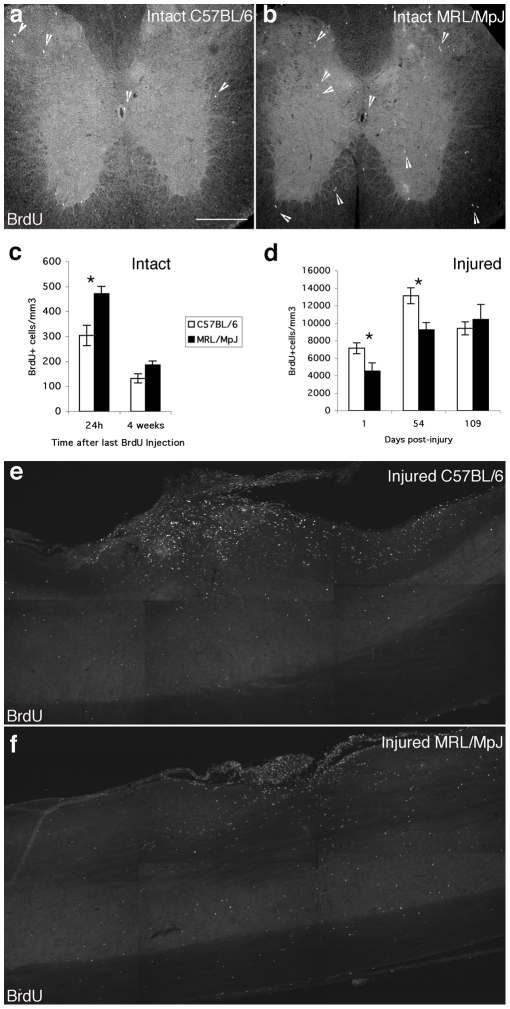
Higher cell proliferation in intact spinal cord but lower increase in cell proliferation after SCI in MRL/MpJ mice. **a**, **b**, Immunohistochemical staining for BrdU (arrowheads) on cross sections at the mid-thoracic level of intact spinal cord in C57BL/6 (**a**) and MRL/MpJ (**b**) mice 24 h after the last of 6 daily BrdU injections. **c**, Graphs show the amount of BrdU-labeled nuclei per mm^3^ in the intact spinal cord at the thoracic level 24 h and 4 weeks after the last BrdU injection. MRL/MpJ mice showed a 1.5× higher level of cell proliferation than C57BL/6. Cell survival was assessed at 4 weeks and there was no significant difference between the two strains. **d**, Number of BrdU-labeled nuclei per mm^3^ in the injured spinal cord 1, 54 and 109 dpi. BrdU was injected after injury and for the 6 following days. MRL/MpJ spinal cord showed less cell proliferation at 1 and 54 dpi. **e**, **f**, Sagittal sections of the lesion epicenter at 54 dpi stained for BrdU in C57BL/6 (**e**) and MRL/MpJ (**f**) mice. Asterisks denote significant difference between MRL/MpJ and C57BL/6, P<0.05 (Student's t-test). Scale bar, 260 µm.

Quantification of BrdU-positive cells in intact spinal cord 1 day after six days of BrdU application revealed a higher level of proliferation in MRL/MpJ mice (p<0.05; Fig. 4b,c). Survival of the progeny of dividing progenitor cells was assessed by evaluating BrdU-positive cells 4 weeks after the last BrdU injection and comparing the number of labeled cells with the number obtained at 1 day after the last injection. The total number of surviving BrdU-positive cells after 4 weeks in MRL/MpJ spinal cord was not significantly different from C57BL/6 mice (p>0.05; Fig. 4c). The cell fate of the BrdU-positive cells 4 weeks post-injection was also assessed in the intact spinal cord. As reported previously for the rat intact spinal cord [23], there was no sign of ongoing neurogenesis detected in MRL/MpJ or C57BL/6 intact spinal cords. In both strains, the majority of the BrdU-positive cells was immature glia (co-labeled with NG2) or mature astrocytes (co-labeled with S100b/GFAP). No significant differences were detected between MRL/MpJ and C57BL/6 (data not shown).

As described previously [28], [29], [30], a substantial increase in cellular proliferation occurs after SCI in and around the injury site (Fig. 4d–f). We injected BrdU 2 h after SCI and once daily for 6 dpi. When compared to intact mice, the number of proliferating cells at the site of injury at 1 dpi was 23.5-fold higher for C57BL/6 mice and only 9.6 times higher for MRL/MpJ mice (p<0.05), showing that MRL/MpJ injured cord contained 1.5 times fewer BrdU-labeled nuclei than C57BL/6 lesioned cord. The number of BrdU-labeled nuclei in MRL/MpJ mice was still 1.5 times lower at 54 dpi (Fig. 4 e, f), whereas no significant difference was found at 109 dpi.

Apoptotic cells were detected at 1 dpi using TUNEL labeling [31]. Numerous TUNEL-positive cells exhibiting typical fragmented and condensed nucleus were detected at the injury epicenter in both mouse strains (MRL/MpJ: 3292±569 apoptotic cells/mm^3^; C57BL/6 3645±631 apoptotic cells/mm^3^). Similarly, Fluorojade staining specifically detecting neuronal cell death [32] revealed no differences between MRL/MpJ (2499±101 Fluorojade-positive cells/mm^3^) and C57BL/6 mice (2590±348 degenerating neurons/mm^3^) at 1 dpi at the injury site.

### Reduced astrocytic and increased microglial differentiation of proliferating cells in MRL/MpJ injured spinal cord

Since it has been suggested that the astrogliotic scar is in part produced by proliferating precursor cells differentiating into astrocytes post-injury [29], [33], [34], [35], the cell fate of the dividing cells after SCI was examined in both strains.

In intact mice, differentiation of the surviving BrdU-positive cells was examined in the spinal cord 4 weeks after the last BrdU injection by means of confocal microscopy and immunofluorescent double labeling for BrdU with lineage-specific markers (neuronal markers NeuN and TUJ1, glial progenitor marker NG2, astrocyte markers GFAP and S100b, microglia marker OX-42). No significant differences were found between MRL/MpJ and C57BL/6 mice (data not shown). In accordance with previous publications [23], [28], no evidence of neurogenesis was found. Many proliferating cells were oligodendroglial progenitor cells (>40%), approximately 5% were astrocytes and fewer than 1% were microglia.

**Figure 5:**
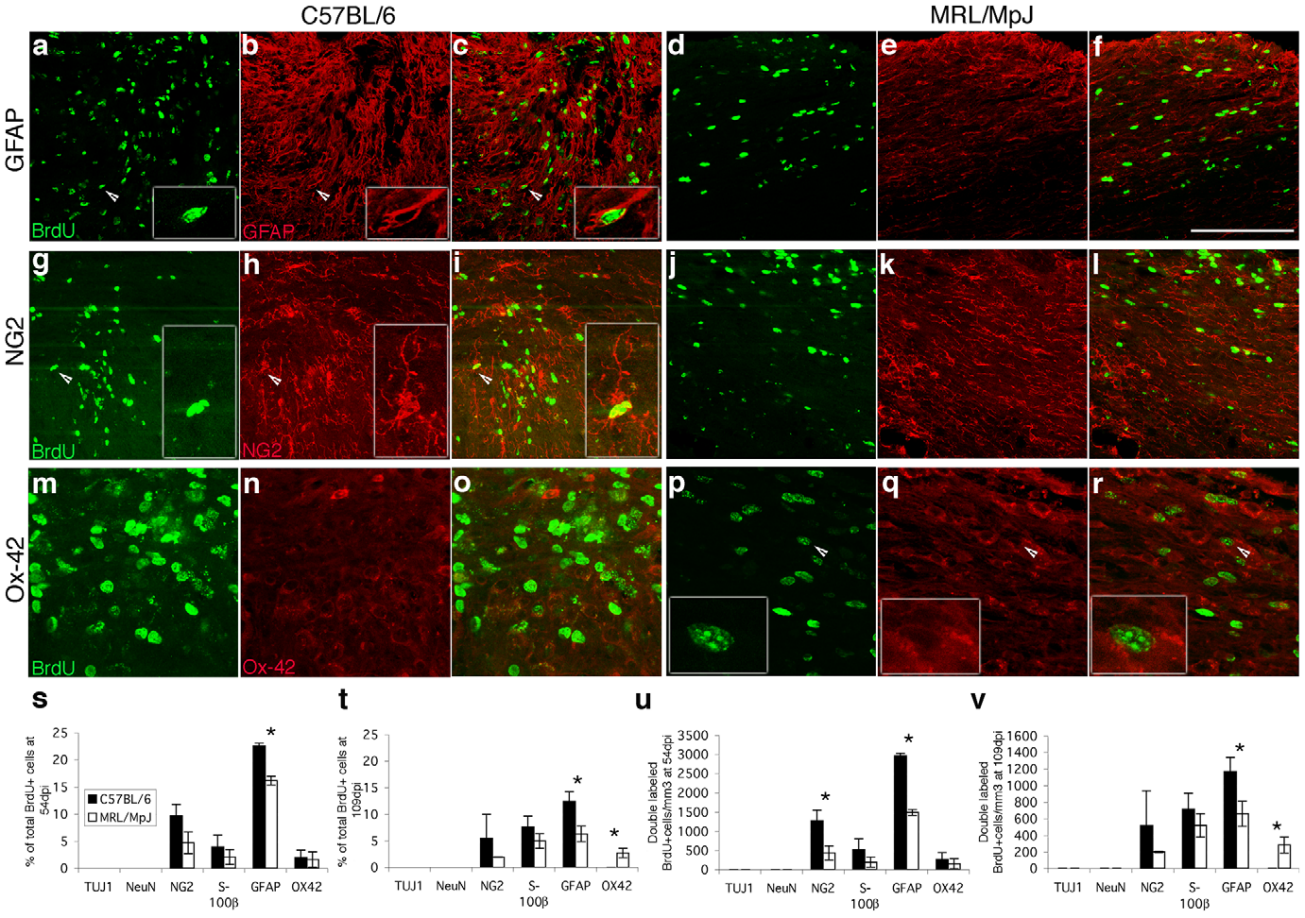
Reduced astrocytic differentiation in MRL/MpJ injured spinal cord. Sagittal sections of the lesion epicenter at 54 dpi (**a**–**l**) and 109 dpi (**m**–**r**) labeled for BrdU (green) and lineage markers (red): GFAP (**a**–**f**), NG2 (**g**–**l**), OX-42 (**m**–**r**) in C57BL/6 (**a**–**c**, **g**–**i**, **m**–**o**) and MRL/MpJ (**d**–**f**, **j**–**l**, **p**–**r**) mice. Higher magnifications of cells marked by the arrowheads are shown in boxed inserts. **s**–**v**, Graphs represent the quantification of BrdU-labeled cells stained for a specific marker at 54 dpi (**s**, **u**) and 109 dpi (**t**, **v**) shown as percentage of all BrdU-labeled cells (**s**, **t**) and as absolute number of co-stained BrdU-labeled nuclei per mm^3^ (**u**, **v**). At 54 dpi, C57BL/6 mice showed more NG2/BrdU- and GFAP/BrdU-positive cells than MRL/MpJ mice. At 109 dpi, C57BL/6 mice had still more GFAP/BrdU-expressing cells than MRL/MpJ mice; however, OX-42/BrdU-positive cells were only found in MRL/MpJ spinal cord (2.7±0.9%). Asterisks denote significant difference between MRL/MpJ and C57BL/6, P<0.05 (Student's t-test). Scale bar, 100 µm (**a**–**i**); 50 µm (**p**–**r**); 50 µm for boxed area (**a**–**i**); 25 µm for boxed area (**p**–**r**).

We then assessed the phenotype of BrdU-positive cells in injured mice. At 54 dpi, no neurogenesis occurred in either of the strains and most of the surviving proliferating cells exhibited a glial phenotype (Fig. 5). The proportions of BrdU-positive cells co-labeled with NG2 (9.7±2.1% vs 4.7±2.0%), S100b (4.0±2.1% vs 2.9±1.4%) and OX-42 (2.0±1.4% vs 1.6±1.4%) in C57BL/6 versus MRL/MpJ mice were not significantly different (Fig. 5s). Nevertheless, BrdU+/GFAP+ cells were significantly higher in C57BL/6 mice (22.5±0.5%) than in MRL/MpJ animals (16.1±0.8%; p<0.05; Fig. 5s). When we assessed double-labeled cells (absolute numbers) per volume, we found that C57BL/6 mice had almost 3 times more NG2+/BrdU+ cells (Fig. 5g–l) and 2 times more GFAP+/BrdU+ cells (Fig. 5a–f) than MRL/MpJ mice (Fig. 5u; p<0.05), but there was no significant difference between the number of OX-42+/BrdU+ cells. At 109 dpi, the phenotype of BrdU-labeled cells was similar to 54 dpi with C57BL/6 mice, showing still more GFAP+/BrdU+ cells than MRL/MpJ mice (1167.8±172.3 vs 660.9±151.5; p<0.05; Fig. 5t, v). But interestingly, at 109 dpi, OX-42+/BrdU+ cells were absent in C57/BL6 mice, whereas they represented 2.7±0.9% of the MRL/MpJ BrdU-labeled cells (p<0.05; Fig. 5n–r, t, v).

### Gene expression profiling of the MRL/MpJ and C57BL/6 injured spinal cord

**Figure 6:**
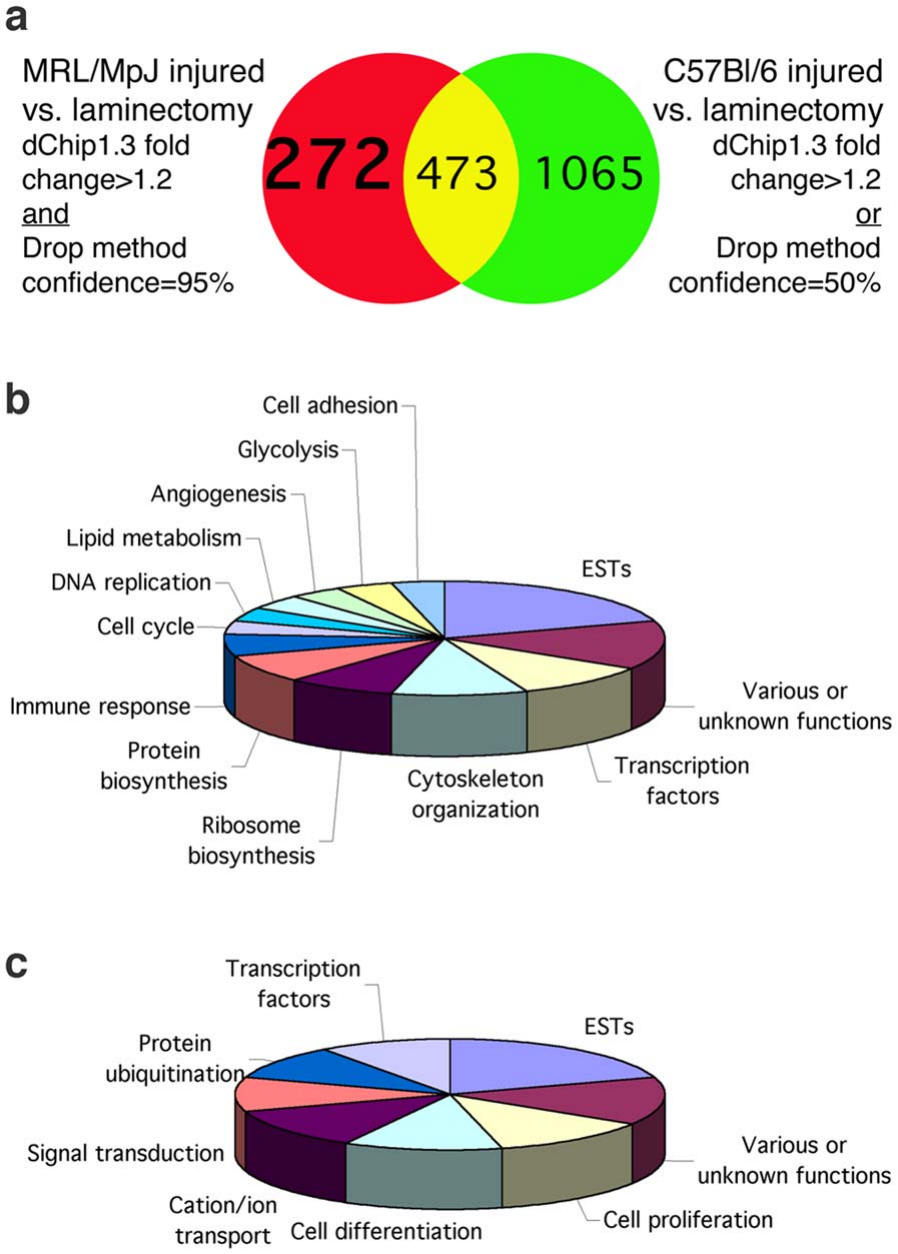
Gene expression profiling of the MRL/MpJ and C57BL/6 injured spinal cord. **a**, Using loose criteria (dChip1.3 fold change>1.2 OR Drop method confidence = 50%), 1538 genes were found to be differentially expressed in C57BL/6 spinal cord 4 days after hemisection. Using strict criteria (dChip1.3 fold change>1.2 AND Drop method confidence = 95%), 745 differentially expressed genes were found in MRL/MpJ spinal cord 4 days after hemisection, and 272 of these genes were exclusive for the MRL/MpJ injured spinal cord. **b**, Classification by biological function of genes up-regulated exclusively in MRL/MpJ injured spinal cord. **c**, Classification by biological function of genes down-regulated exclusively in MRL/MpJ injured spinal cord.

To assess the molecular events occurring in the MRL/MpJ spinal cord after injury and to reveal putative candidate genes responsible for the enhanced spinal cord repair, we used microarray technology. To exclude as many genes as possible that are changed by SCI in general, and to find genes that are strictly differentially regulated in MRL/MpJ mice, we used different criteria when analyzing the gene chips. We used “loose” criteria (dChip1.3 fold change>1.2 OR Drop method confidence = 50%) to reveal differentially expressed genes in C57BL/6 spinal cord 4 days after dorsal hemisection and found 1538 genes. We applied “strict” criteria (dChip1.3 fold change>1.2 AND Drop method confidence = 95%) to find genes differentially expressed in MRL/MpJ spinal cord 4 days after dorsal hemisection and found 745 genes. Of these genes, 272 are exclusively regulated in MRL/MpJ injured spinal cord (Fig. 6a; complete data set available at http://genechip.salk.edu/rawdata/Thuret_MRL.zip). We also compared gene expression in the intact spinal cord of MRL/MpJ and C57BL/6 mice using the “strict” criteria and found 54 genes to be differentially expressed. None of these genes was among the 272 genes that were differentially expressed in MRL/MpJ injured spinal cord exclusively.

We focused our interest on the genes that were exclusively differentially expressed in MRL/MpJ injured spinal cord and classified the 103 upregulated genes (Fig. 6b) and the 169 downregulated genes by biological function (Fig. 6c). More than a quarter of these genes are to date Expressed Sequence Tags (ESTs) or have an unknown function. Following SCI, MRL/MpJ mice appear to upregulate mainly genes implicated in repair mechanisms: 10% of the upregulated genes are involved in cytoskeleton reorganization, 16% take part in protein and ribozome biosynthesis and 24% participate in cell cycle, DNA replication, lipid metabolism, angiogenesis, glycolysis or cell adhesion. Genes involved in inflammatory processes (6%) are also upregulated upon SCI in MRL/MpJ mice. Among the 169 down-regulated genes, 24% are involved in cell proliferation and differentiation, 12% in cation/ion transport and 12% take part in the signal transduction cascade. Genes participating in the protein ubiquitination process are also downregulated (10%), suggesting a diminished cellular degradation upon SCI in MRL/MpJ mice. Table 1 shows the 10 most up- and down-regulated genes in MRL/MpJ injured spinal cord of which expression levels have also been verified by Q-PCR. In particular, the interferon activated gene 202B (*Ifi202b*) -encoding p202b [36]- is increased 35.66 fold in the injured MRL/MpJ spinal cord and is unchanged in C57BL/6 (Table 1). The second highest fold change difference (x7.59) is the allograft inflammatory factor 1 gene (*Aif1*). This gene is an interferon (IFN)-gamma-inducible Ca2+-binding EF-hand protein.

The identification of candidate genes that complement our histological findings reveals potential molecular mechanisms associated with the enhanced spinal cord healing ability of MRL/MpJ mice.

## Discussion

The aim of this work was to assess whether MRL/MpJ mice show enhanced spinal cord repair upon injury. We performed a dorsal spinal cord hemisection and used the C57BL/6 mouse as a control strain. We show in this study that MRL/MpJ mice recover faster than C57BL/6 mice in several behavioral tests. This recovery parallels the enhanced regeneration of CST axons in MRL/MpJ mice. Our data suggest that these axons have a facilitated passage across the injury site because of the reduced astrocytic response and diminished micro-cavity formation in MRL/MpJ mice. The reduced astrocytic response is probably due to reduced cell proliferation post-injury and reduced astrocytic differentiation of the proliferating cells. In addition, we observed an increased microglial differentiation in MRL/MpJ mice, suggesting a possible role of the inflammatory response in the MRL/MpJ spinal cord repair mechanisms. Finally, to evaluate the molecular basis of enhanced spinal cord repair, we examined the gene expression changes after SCI in MRL/MpJ and C57BL/6 mice. The microarray data complement our histological findings and unveil a transcriptional profile that reflects the more efficient spinal cord repair in MRL/MpJ mice

### Locomotor recovery and axonal regeneration

The dorsal hemisection destroys a portion of supraspinal input to the lumbar spinal cord and produces major, but incomplete, loss of hindlimb function. Moreover, there is a slight possibility for a minority of CST fibers to not have been fully transected in our model. The limited recovery of C57BL/6 animals in motor tasks may be attributed to distal rearrangements of spared descending fibers. Recently, it has been demonstrated that local plasticity after SCI creates new circuits between descending CST fibers and cervico-lumbar propriospinal neurons [37]. In MRL/MpJ mice, the recovery of locomotor function was significantly improved. Only in the MRL/MpJ mice did we find CST fiber growth caudal to the injury site, suggesting an important role for regrowing fibers in behavioral recovery. CST axonal regeneration, similar to what we observed in MRL/MpJ mice, has been associated with functional recovery after treatment and in transgenic mice [8], [38], [39], [40], [41], [42]. It is also possible that the inherent plasticity of the injured spinal cord for creating new circuitries [37] is enhanced in MRL/MpJ mice. We assume that MRL/MpJ CST axons grow because of a more permissive environment at the injury site: (i) we observed fewer micro-cavities providing a better environment for tissue repair and axonal regeneration [43], [44] and (ii) we found fewer astrocytes at the injury site, which have been shown to form a physical barrier preventing axonal growth [7]. Thus, we suggest that MRL/MpJ mice have a more permissive environment for axonal regeneration, with a reduced physical barrier and probably a diminished production of growth-inhibitory molecules.

### Astrocytic response

For both MRL/MpJ and C57BL/6 mice, the majority of proliferating cells at the injury site differentiated into astrocytes. However, C57BL/6 mice had twice as many newborn astrocytes than MRL/MpJ. Astrocytes are the major contributor to the growth-inhibitory scar after an injury, which is one of the obstacles for successful regeneration. It has been suggested that part of the astrogliotic scar formed after injury is attributable to newly generated astrocytes and not to activation or migration of resident astrocytes [33]. MRL/MpJ injured spinal cord contains fewer newborn astrocytes, probably due to (i) a diminished increase in cell proliferation at the injury site and (ii) a lower astrocytic differentiation of the proliferating cells. X-irradiation treatment eliminates rapidly dividing cells after SCI, suppresses glial scar formation [9], [45] and improves functional recovery [9], [46]. Taken together, these data suggest that limiting cell proliferation upon SCI might be beneficial to reducing astrogliosis and the formation of scar tissue.

### Inflammatory response

Our data from the gene profiling analysis at 4 dpi suggest an amplified inflammatory response in injured MRL/MpJ spinal cord. Among the genes exclusively differentially expressed in MRL/MpJ injured spinal cord, the second highest fold change difference (x7.59) is the allograft inflammatory factor 1 gene (*Aif1*; Table 1). This gene encodes an interferon (IFN)-gamma-inducible Ca2+-binding EF-hand protein. In the brain, a subset of microglial cells constitutively expresses AIF-1 [47]. Increased numbers of AIF-1-immunoreactive macrophages/microglial cells were observed in brain tumors and ischemia [48] as well as after SCI [49].

The spinal cord tissue analysis at 109 dpi showed a higher rate of microglia differentiation and/or macrophage recruitment in MRL/MpJ mice, indicating that the immune response might be sustained for the entire repair process. Immune system activity has traditionally been considered harmful for recovery after SCI [48], [50], [51], [52]. However, some studies have shown the potential beneficial effect of a strong inflammatory response. For example, Franzen and colleagues [53] have transplanted macrophages into the injured adult rat spinal cord and suggest that macrophages may exert beneficial effects by degrading myelin products, which inhibit axonal re-growth, and by promoting a permissive extracellular matrix. Moreover, Rapalino and colleagues [54] observed partial recovery of paraplegic rats after implantation of stimulated homologous macrophages. Lastly, Foote and Blakemore [55] have shown that inflammation stimulates remyelination in areas of chronic demyelination. The increased immune response seen in the gene profiling analysis might be due to a more permeable blood-brain-barrier (BBB) because of reduced gliosis. Furthermore, MRL/MpJ mice contain elevated numbers of macrophage progenitor cells circulating in their blood [56], which could possibly pass the more permeable BBB, invade the injury site and proliferate.

Interestingly the Transforming Growth Factor beta is a key regulator in immune responses [57] and its mRNA is upregulated 1.9-fold more in MRL/MpJ injured spinal cord. Injecting TGFb1 was shown to stimulate astrocytes [58]; however, a recent study found an exacerbated astroglial reaction in TGFb1−/− mice [59]. The spinally injured MRL/MpJ mice in our studies showed a reduced astroglial reaction and decreased micro-cavity formation. Importantly, TGFb1 was shown to promote the survival of various CNS neurons *in vitro*, including motoneurons [60]. Additionally, TGFb1 prevents neuronal degeneration caused by hypoxic or excitotoxic injury *in vitro* and rescues hippocampal CA1 neurons from death after transient ischemia *in vivo* [61], [62]. Furthermore, anti-TGFb1 treatment following SCI exacerbates secondary damage by increasing the size of cavities in the injured tissue [63], and treatment of SCI rats with TGFb1 reduces lesion volume [64].

The inbred MRL/MpJ strain has a mild autoimmune disorder and is used as a background strain for mutating the Fas gene leading to lupus erythematosus (SLE). The severity of autoimmunity in these mutant mice is strongly affected by the genetic background in which they are placed [65]. The inbred MRL/MpJ strain background creates a severe lupus disease, and even *Fas*-sufficient MRL/MpJ mice suffer from a mild-late-onset SLE [66]. Interestingly, recent evidence suggests that autoimmunity is an endogenous response to CNS injury and that it can be beneficial for repair [67], [68]. Further studies showed that passive transfer of T-cells specific to myelin basic protein reduces neuronal loss after SCI and promotes recovery in rats [69], [70], [71]. Together these data suggest that part of MRL/MpJ functional recovery might be linked to their mild autoimmune disorder.

### Brain repair versus spinal cord repair

Hampton and colleagues [72] studied the brain response to injury in MRL/MpJ mice. They did not observe enhanced axonal regeneration after cutting the dopaminergic projection from the substantia nigra to the striatum. Neither did they see a reduced scar after a stab lesion to the cortex. The only common observation between this and the present study was the enhanced and prolonged microglial activity.

In contrast to what we found in the spinal cord, they observed a higher increase in cell proliferation in the cortex after injury. Interestingly, in MRL/MpJ spinal cord we found a dramatic +35.88-fold change for a gene whose over-expression in mammalian cells inhibits cell proliferation: the interferon activated gene 202B (*Ifi202b*) encoding the p202b protein. This inhibition of proliferation might be correlated with the binding and inhibition of the activity of several transcription factors by p202, such as c-Fos, c-Jun, AP2, E2F-1, E2F-4, MyoD, myogenin, and NF-kB p50 and p65 [73], [74], [75].

In unlesioned MRL/MPJ mice, we observed a lower cell proliferation in the hippocampus than in C57BL/6 animals [76], whereas in the uninjured MRL/MpJ spinal cord we observed a higher cell proliferation. Proliferation in the brain and the spinal cord are differently regulated in intact tissue, therefore it might be also the case following injury. In general, spinal cord and brain seem to react conversely regarding cell proliferation: running increases cell proliferation in the hippocampus but decreases proliferation in the spinal cord of C57BL/6 (our unpublished data).

Another disparity between brain and spinal cord involves the inflammatory response. The acute inflammatory response to traumatic injury is significantly greater in the spinal cord than in the cerebral cortex [77]. Overall, we suspect that the disparity between MRL/MpJ brain and spinal cord repair might be due to a combination of intrinsic differences in cell proliferation and inflammatory response.

### Hemisection versus Contusion

More recently, Kostyk and colleagues [78] have assessed the spinal cord regenerative abilities at T9 of MRL/MpJ versus C57/Bl6 mice. They also reported a robust axon growth within the lesion at 28 dpi in MRL/MpJ mice. However, the extent of locomotor recovery was reported to be impaired in the MRL/MpJ mice by 42 dpi. They further described evidence of ongoing degeneration both within and around the lesion site at 42 dpi. Interestingly, Kostyk and colleagues have used a contusion model using rapid displacement of an impounder on the exposed spinal cord following a dorsal laminectomy producing a contusion of moderate severity. These data are consistent with our initial pilot contusion data using the IH-impactor device at 50Kdynes (data not shown), which lead to impaired locomotor recovery in MRL/MpJ mice by 43 dpi. This is in contrast to the present study using a dorsal hemisection model where axonal growth was consistent with functional recovery. It is important to highlight that to inflict a contusion injury using an impounder, it is necessary to make a very large laminectomy. This is in contrast with making a very small laminectomy for a dorsal hemisection. Interestingly, postmortem dissection of our contused mouse samples (data not shown) uncovered a cartilaginous overgrowth within the large laminectomy region inducing a fusion between the remaining neighboring vertebrae leading to extreme spinal column distortion. This distortion possibly explains the poor motor performance of this contusion group. In contrast, such cartilaginous overgrowth was never observed with small laminectomies associated with the dorsal hemisection procedures.

Moreover, a contusion injury is a crude injury as opposed to a micro-scissors hemisection, which is a sharp injury. Interestingly, it has been shown that regeneration of the ear in MRL/MpJ mice depends on the type of wound trauma, where a crude injury (punch) does not heal as well as a sharp injury (clinical biopsy) [79]. Indeed, Rajnoch and colleagues [79] have previously shown that in crude thumb-punched MRL/MpJ ears, an increase in wound area diameter was still observed at the day-28 time point. By contrast, biopsy-punched MRL/MpJ ears showed an 85% wound closure over the same period.

The observations of this study, together with Hampton and colleagues [72] as well as Kostyk and colleagues [78] findings suggest that the occurrence of an enhanced regenerative ability is not exclusively dictated by genetic influences, but is also highly dependent on local cellular interactions and injury type.

### Validation of the gene expression profiling after SCI

To help us understand the events occurring in the MRL/MpJ spinal cord after injury, we analyzed gene expression changes after SCI in both strains and compared the results to compile a list of genes differentially expressed exclusively in MRL/MpJ injured spinal cord. Since it is known that genes are differentially regulated in different strains of mice or rats [80], [81], we only focused on genes that are specifically expressed in injured MRL/MpJ spinal cord. We have evidences that the MRL/MpJ differentially expressed genes identified in this screen are potentially implicated in spinal cord repair. First, we observed, in both strains some genes that have previously been shown to be differentially regulated after SCI. For example, both mouse strains show expression changes in genes coding for proteins that influence apoptosis: Caspase 3 is upregulated and Caspase 7 is downregulated, as observed previously [25], [82]. These studies also found Bax and Bak1 to be upregulated upon SCI, as we have in the present study. For both injured strains, genes coding for cathepsin proteases are upregulated (cathepsins B, C, D, L and S), as previously reported [25], [83], [84], [85]. These data validate that our method is useful for identifying candidate genes that contribute to differences in spinal cord repair between MRL/MpJ and C57BL/6 mice. Second, we found that some of the genes differentially expressed in MRL/MpJ injured spinal cord are located on the quantitative trait loci QTLs that control ear healing in MRL/MpJ mice [16], [19], [21]. These studies found linkage on chromosome 7. Interestingly, some of the genes differentially expressed in injured MRL/MpJ spinal cord (e.g., genes coding for the mitochondrial uncoupling protein 2 and TGFb1) are located in or near the loci on chromosome 7. Additionally, Masinde et al. [21] found linkage on chromosome 1, where Ifi202b, another of our candidate genes, is located. We found that some genes differentially expressed in the MRL/MpJ healing ear are also present on our gene list. For example we observed that *Decorin* is less up-regulated whereas *DEAD/H* and *Proteasome (prosome, macropain)* are more up-regulated in MRL/MpJ, as previously reported in a different lesion model [21]. We also found that *integrin beta2* and *secretory granule proteoglycan* are more up-regulated in MRL/MpJ upon injury, as shown previously [20]. Since rapid wound healing in MRL/MpJ mice is a genetically controlled quantitative trait [15], [19], we expected that the soft tissue regeneration QTLs and genes found in studies on ear regeneration in MRL/MpJ mice would overlap with the genes discovered in this study. The fact that some of the differentially expressed genes are located within the QTL regions or have already been identified provides another validation of our screen. Third, our microarray data complement our histological findings. Indeed, the general tendency is the up-regulation of genes involved in repair mechanisms. Moreover, genes involved in lowering cell proliferation (e.g., *Ifi202b*) and increasing the immune response (e.g., *Aif-1*) are up-regulated in the injured MRL/MpJ spinal cord.

We recognize that this study may underestimate the number of genes involved in MRL/MpJ spinal cord healing because (i) we examined only one time point and some changes in the expression may occur at an earlier or later stage of the repair process, (ii) we used only one strain of mice for control, and (iii) we collected only the lesion epicenter and additional genes might be differently regulated rostral and/or caudal to the lesion. Moreover, it would be interesting in future studies to extract RNA from specific cell types to identify the particular cells responsible for these significant gene expression changes. However, the genes identified here may play a role in the repair processes following SCI and further characterization of these candidates might elucidate some molecular mechanisms of spinal cord repair.

### Conclusion and perspectives

This study shows that the MRL/MpJ mice are able to recover faster and better after spinal cord dorsal hemisection than C57BL/6 mice. Our data suggest that a sustained immune response and decreased astrocytic activity are likely to be part of a complex recovery course. Although our data are not able to explain the detailed mechanisms of such a recovery, they do demonstrate that the repair process after SCI is multifaceted. Many pathways are involved, all of which need to be considered and each of which needs to work at an optimal “level” to result in behavioral recovery.

## Supporting Information

Table S1 **Primer Sequences for Q-PCR.**
(DOCX)
