# Supplementary material for: Enhanced Functional Recovery in MRL/MpJ Mice after Spinal Cord Dorsal Hemisection
Source: PLoS One. 2012 Feb 13;7(2):e30904. doi: 10.1371/journal.pone.0030904 (PMC3278405; doi:10.1371/journal.pone.0030904)
Supplement: Table S1 — Primer Sequences for Q-PCR. (DOCX) [file pone.0030904.s001.docx]

Table S1. Primer Sequences for Q-PCR

| **Mouse Gene** | Forward primer sequence (5'-3') | Reverse primer sequence (5'-3') |
| --- | --- | --- |
| interferon activated gene 202B  NM_008327.2 | GAAGTTCCCGGCTTGAAGAACTCA | GCAGCCTCTGACACAGTGGCA |
| allograft inflammatory factor 1 NM_145144.**1** | GCCGGAAAAGCTCGCAGCCT | CGACCGCTTGCCCAGCATCA |
| histidine ammonia lyase  NM_010401.3 | AGCGGAGACCTTGCCCCACT | TTGCCCACGGTGAGGTCGGA |
| apolipoprotein B editing complex 1  NM_009694.3 | GCTTTCGACCCGGCCCTCAA | GCGAAGTTTGCAGCCGGCCT |
| Rho, GDP dissociation inhibitor (GDI) beta  NM_007486.4 | ACTGGCGATCTCGAGGCCCTC | TACTCCTCTGGTCGGGGCCC |
| beta-glucuronidase  NM_010368.1 | CTGCTCTGAAACCCGCCGCA | GCGGGTCCTCGTGGATCCCT |
| cysteine rich protein 61  NM_010516.2 | CCTCCTGCGCGCCACAATGA | GGTGGAGCTGGCGCCGAAAT |
| potassium voltage-gated channel, shaker-related subfamily, beta member 1  NM_010597.3 | GTGGAGGTCCAGCTGCCGGA | CTCAGGCACCACGCCACAGC |
| calcitonin-related polypeptide, beta  NM_054084.2 | TCGCTGTTCCAACACGGGCTAG | GGCTGCTCTCCAAAGCGGACC |
| vesicle-associated membrane protein 1  NM_001080557.**1** | CCTTGCAGGCCGGAGCATCAC | TCCCTCCCAAACCAGACGACGAT |
| sulfotransferase family 4A, member 1  NM_013873.3 | GTCTACAGCAGCCGCCTGGC | ACGGCCTGGGACAGGTCCTG |
| regulator of G-protein signaling 4  NM_009062.3 | AGCCAATGTACCGGGCTGCA | CATGGACCAGCGGCAGGCAG |
| Parvalbumin  NM_013645.3 | GTCCTTCTCGCGGCCCTCCT | AACCAACACCCTGCCAGGCC |
| creatine kinase, mitochondrial 1, ubiquitous  NM_009897.2 | CGTCCTCTCTCTACCCGGTTGCA | TCAGCGCTCGGGGGATACAGT |
| cartilage acidic protein 1  NM_145123.4 | ATCGATGGGGACGGCCGTGA | CCCGGCCCGCTGTGTACTTG |
| solute carrier family 17 (sodium-dependent inorganic phosphate cotransporter), member 6  NM_080853.3 | GACTGCACGTGCTTCGGCCT | CAGCCAGCCGCGATGCGATA |
